# *N*,*N*-Dimethyl-3β-hydroxycholenamide attenuates neuronal death and retinal inflammation in retinal ischemia/reperfusion injury by inhibiting Ninjurin 1

**DOI:** 10.1186/s12974-023-02754-5

**Published:** 2023-04-07

**Authors:** Yunhong Shi, Yidan Liu, Caiqing Wu, Xiuxing Liu, Wenfei Hu, Zhenlan Yang, Zhidong Li, Yangyang Li, Caibin Deng, Kun Wei, Chenyang Gu, Xuhao Chen, Wenru Su, Yehong Zhuo

**Affiliations:** State Key Laboratory of Ophthalmology, Zhongshan Ophthalmic Center, Guangdong Provincial Key Laboratory of Ophthalmology and Visual Science, Sun Yat-Sen University, No. 7 Jinsui Road, Tianhe District, Guangzhou, 510060 Guangdong China

**Keywords:** Ischemia–reperfusion injury, Single-cell RNA sequencing, Neuroprotection, Inflammation, Nerve injury-induced protein 1

## Abstract

**Background:**

Retinal ischemia–reperfusion (RIR) injury refers to an obstruction in the retinal blood supply followed by reperfusion. Although the molecular mechanism underlying the ischemic pathological cascade is not fully understood, neuroinflammation plays a crucial part in the mortality of retinal ganglion cells.

**Methods:**

Single-cell RNA sequencing (scRNA-seq), molecular docking, and transfection assay were used to explore the effectiveness and pathogenesis of *N,N*-dimethyl-3β-hydroxycholenamide (DMHCA)-treated mice with RIR injury and DMHCA-treated microglia after oxygen and glucose deprivation/reoxygenation (OGD/R).

**Results:**

DMHCA could suppress inflammatory gene expression and attenuate neuronal lesions, restoring the retinal structure in vivo. Using scRNA-seq on the retina of DMHCA-treated mice, we provided novel insights into RIR immunity and demonstrated nerve injury-induced protein 1 (Ninjurin1/Ninj 1) as a promising treatment target for RIR. Moreover, the expression of Ninj1, which was increased in RIR injury and OGD/R-treated microglia, was downregulated in the DMHCA-treated group. DMHCA suppressed the activation of the nuclear factor kappa B (NF-κB) pathways induced by OGD/R, which was undermined by the NF-κB pathway agonist betulinic acid. Overexpressed Ninj1 reversed the anti-inflammatory and anti-apoptotic function of DMHCA. Molecular docking indicated that for Ninj1, DMHCA had a low binding energy of − 6.6 kcal/mol, suggesting highly stable binding.

**Conclusion:**

Ninj1 may play a pivotal role in microglia-mediated inflammation, while DMHCA could be a potential treatment strategy against RIR injury.

**Supplementary Information:**

The online version contains supplementary material available at 10.1186/s12974-023-02754-5.

## Background

Ischemia–reperfusion injury, a well-known pathologic hallmark associated with multiple degenerative diseases, refers to a reduction in the blood supply and then the oxygen and various nutrients; subsequent blood reperfusion induces oxidative stress and inflammatory responses [1]. Ischemia in the retinas has been extensively studied due to its impact on glaucoma, diabetic retinopathy, traumatic optic neuropathy, central retinal venous occlusion, and retinopathy of prematurity, among others [2]. Retinal Ischemia–reperfusion (RIR) injury pathogenesis is characterized by a self-reinforcing destructive cascade, such as the dysfunction of mitochondria; oxidative stress; activation of glial cells, and retinal pigment epithelium; ultimately terminating in photoreceptor cell death [3]. Owing to the relative lack of effective treatment options, RIR remains a major challenge of blindness worldwide [4].

Inflammation has been widely recognized as a common characteristic of RIR injury, and its extent and persistence affect the endpoint pathology, i.e. neuronal death [5]. Following RIR injury, astrocytes, Müller cells, and microglia produce mediators such as cytokines and interleukins [6]. It has been reported that in microglia, as the potential cellular regulators of inflammation, activation is the pilot event during a neural injury [7]. Microglia exhibit various phenotypes involved in inflammation [8, 9] by secreting two pleiotropic cytokines, namely tumour necrosis factor-α (TNFα) and interleukin (IL)-1β, which facilitate the recruitment of leukocytes into the retina [10]. In view of this, therapies aimed at modulating microglia's inflammatory responses and immune homeostasis represent a promising approach [11]. We have recently shown that programmed cell death (PCD), including apoptosis, necroptosis, pyroptosis, and ferroptosis, can induce severe inflammation in RIR injury [12, 13]. Hence, an in-depth investigation of the molecular pathobiology underlying RIR-induced neuroinflammation, retinal ganglion cell (RGC) death, and identification of new therapeutic targets are desperately needed.

The *nerve injury-induced protein 1* gene (*Ninjurin 1* or *Ninj 1*) encodes a cell surface protein of 152 amino acids that contains two transmembrane domains [14]. The Ninj1 protein was initially found to concentrate in dorsal root ganglion neurons and Schwann cells and mediate homophilic contacts, thereby suggesting its potential involvement in nerve regeneration [14]. The importance of oligomerized Ninj1 in generating plasma membrane rupture, a critical pyroptotic event, was emphasized [15]. This function may be carried out via the putative-helix domain of the protein. Additionally, research has demonstrated that pyroptotic cells emit certain intracellular damage-associated molecular patterns (DAMPs) in a Ninj1-dependent manner, indicating the pro-inflammatory activity of Ninj1 [16]. Ninj1 is also widely expressed in immune cells, including macrophages, microglia, and monocytes, as well as in hepatocellular carcinoma, fibroblasts, epithelial cells, pericytes, and other tissues [17, 18]. This suggests that Ninj1 may play a role in the functioning of many tissues [17, 18]. We hypothesize that Ninj1 may contribute to the progression of RIR injury based on the well-known idea that neuroinflammation plays a crucial role in the progression of programmed RGC death in RIR [2, 9].

*N,N*-Dimethyl-3β-hydroxycholenamide (DMHCA) is an experimental synthetic steroid acting as a liver X receptor (LXR) activator. LXRs (LXRα and LXRβ) belong to the nuclear receptor superfamily. Previous studies have shown that LXRs play a critical role in reversing cholesterol transport, regulating immune cell function, and influencing macrophage polarization [19]. According to a recent study, DMHCA reduces the levels of pro-inflammatory M1-like macrophages and classical monocytes, which are involved in diabetic retinopathy's inflammation [20]. Additionally, DMHCA was thought to be a viable therapeutic drug against neurodegenerative conditions like Alzheimer's disease because of its ability to target rat cortical neurons and stop memory loss [21]. However, whether DMHCA mediates neuroprotection or hammering inflammation in RIR injury remains unknown.

The retina ganglion cells, an extension of the central nervous system (CNS), are the most metabolically active place in the body [22]. This makes the retina an ideal model system for research to assess different therapeutic strategies in vivo and effectively extrapolate the findings to the whole body. Therefore, this study explores the effects, potential therapeutic targets, and molecular mechanisms of DMHCA treatment in vivo, using an RIR mice model, and in vitro, focusing on microglia-induced inflammation.

## Methods

### Animals

The 6- to 8-week-old wild-type C57BL/6J mice (male, RRID:IMSR_JAX:005304) were purchased from Guangdong Medical Laboratory Animal Center and raised in the Experimental Animal Center of Zhongshan Ophthalmic Center, Sun Yat-sen University. All animal experiments were approved by the Animal Care and Ethics Committee of the Zhongshan Ophthalmic Center (ref no. 2015–108, Approval number: O2021080).

### Pharmacologic treatment and RIR injury model

Mice were first dosed with 0.08 g/kg DMHCA daily via oral gavage for three days. The sham and RIR groups received an aqueous solution of 0.9% saline at the same time. Three days after DMHCA treatment, the mice underwent surgical RIR injury as previously described [12, 23]. Briefly, a sterile needle linked to an elevated normal saline (NS) bottle was placed into the anterior chamber of the eyeball to create a persistent intraocular pressure (IOP) at approximately 110 mmHg for 1 h. The needle was gently pulled out to induce reperfusion injury. Three days after reperfusion, the eyeballs or retinal tissues were harvested for analysis.

### Haematoxylin and eosin (HE) staining

Mice's eyes were fixed with 4% paraformaldehyde (PFA) and embedded in paraffin; 7 μm sections were prepared across the optic nerve head of each eye and stained with HE. Four cross-sectional around the optic nerve (within 1000 μm) for every eyeball were chosen to measure the inner plexiform layer (IPL) thickness, and data were analysed using the ImageJ software (version 1.53v, https://imagej.nih.gov/ij/).

### Serum analyses

For serum collection, mice were anaesthetized with pentobarbital sodium (0.01 g/mL), and blood was gathered by cardiac puncture. Serum was separated from clotted blood at 4° via centrifugation at 1200 × *g* for 30 min to separate the serum. Serum was isolated and analysed for triglycerides (TG), total cholesterol (TC), high-density lipoprotein (HDL) cholesterol, and low-density lipoprotein (LDL) cholesterol concentrations using commercial enzymatic kits (JINGME, Jiangsu, China).

### Simulation of in vitro ischemia with oxygen-glucose deprivation/reoxygenation (OGD/R) and microglia pharmacologic treatments

The BV2 microglial cell line was purchased from Zhong Qiao Xin Zhou Biotechnology Company (Shanghai, China). DMHCA (5 μmol/mL) was prepared into the medium 8–12 h before OGD/R. To simulate the model of RIR in vivo, BV2 cells were cultivated in serum- and glucose-free media and placed under conditions of 5% CO_2_ and 95% N_2_ in a 37 °C incubator for 3 h, then returned to the normal environment (37 °C, 5% CO_2_ and normoxic) and complete medium (DMEM with 10% foetal bovine serum and glucose [4.9 g/L]) for 24 h until harvesting.

### Expression vector cloning and transfection

Ninj1(m) cDNA, including coding sequences with or without 3′-untranslated region, was amplified via polymerase chain reaction (PCR) with the forward primer (5′–3′) CTTGGTACCGAGCTCGGATCCGCCACCATGGAGTCGGGCACTGAGGAGTATGAGC and the reverse primer (5′–3′) GAAGGGCCCTCTAGACTCGAGCTGCCGGGGCGCCACGTCCATTACAGGCT. The PCR products were inserted into the pcDNA3.1-3xFlag-T2A-EGFP expression vector (MHBIO, GuangZhou, China) between the EcoRI and XbaI sites (Additional file 1). When BV2 microglia reached 30–50% confluency, they were transfected with Ninj1 plasmids (20 nM) or negative control plasmids (20 nM) using LipoTrans™ Liposomal Transfection Reagent (MHBIO, GuangZhou, China) according to the instruction book.

### Immunofluorescence staining

The eyeballs were embedded in optimal cutting temperature (OCT) compound, frozen, and sectioned at 10 μm. Rabbit polyclonal RBPMS (GeneTex Cat# GTX118619, RRID:AB_10720427) at a concentration of 1:100, beta Tubulin 3/TUJ-1 (GeneTex Cat# GTX130245, RRID:AB_2886220) at a concentration of 1:200, Goat Polyclonal AIF-1/Iba-1 antibody (Novus Cat# NB 100-1028, RRID:AB_521594) at a concentration of 1:100, and Rabbit monoclonal to GFAP N-terminal (Abcam Cat# ab194324) at a concentration of 1:50 were used. DAPI (Bioss Cat# C02-04002) was used for nuclear staining. Images were obtained using immunofluorescence microscope (Leica DMi8, software: Leica Application Suite X Core 3.7.6, leica-microsystems.com).

### Single-cell genomics

#### scRNA-seq library preparation

scRNA-seq libraries were prepared using the Chromium Single Cell 3' Library and Gel Bead Kit (10 × Genomics) according to the manufacturer's instructions. The libraries were sequenced on the NovaSeq platform (Illumina) to yield 150 bp paired-end reads.

#### Analysis of scRNA-seq data

Gene expression matrices were generated using cellranger count, and cellranger aggr in CellRanger (v7.0.0) with default parameter settings by mapping sequencing reads to the 10 mm mouse genome and quantifying the expression of transcripts in each cell. All downstream data processing was conducted using R (v4.1.3) and the Seurat R package (v4.1.1) [24] unless otherwise specified. Cells with more than 200, fewer than 7500 genes, and less than 20% mitochondrial genes were retained for further analysis. Batch effects were removed using the harmony R package (v0.1.0), and subsequent analyses were based on the merged data. The principal components (20 for total cells and myeloid cells, 10 for microglia) were then used to reduce the dimensionality by Uniform Manifold Approximation and Projection (UMAP).

#### Differential expression analysis

Differential expression analysis from different kinds of cells from groups was conducted by "FindMarkers" function of the Seurat R package. Genes with | Log2 (Fold Change) |> 1 and *P* value < 0.05 were established as differentially expressed genes (DEGs).

#### Gene Ontology (GO) and Kyoto Encyclopedia of Genes and Genomes (KEGG) pathway enrichment analysis

Pathway enrichment analyses were completed using the Metascape webtool [25] according to DEGs found between different samples. Five to 10 GO terms or pathways associated with inflammatory responses and visual functions were visualized using the ggplot2 R package (v3.3.6).

#### Inflammatory score

Inflammatory signature scores were calculated using the Seurat function AddModuleScore, which analyses the average expression of interested signatures per cell. This list was created by searching for inflammatory response-related genes (GO: 0006954) found in the DEG datasets between different samples (RIR vs. sham, RIR + DMHCA vs. RIR). Statistical analysis was conducted with the two-sided Wilcoxon rank sum test.

#### Cell–cell communication analysis

Intracellular cell–cell communication was performed with the CellChat R package (v1.4.0) [26]. Differential numbers of interactions and differential interaction strengths were analysed. Cellular pathway networks across the three groups were compared, and the expression of pathway-related genes was visualized using violin plots.

#### Transcription factor (TF)

According to the workflow (http://scenic.aertslab.org/), transcription factor activity was inferred with the pySCENIC package [27].

### Western blotting

The following antibodies were obtained from Cell Signaling Technology: Phospho-nuclear factor kappa B (NF-κB) p65 (Cell Signaling Technology Cat# 3033, RRID:AB_331284), NF-κB p65 (Cell Signaling Technology Cat# 8242, RRID:AB_10859369), Phospho-IKKα/β (Cell Signaling Technology Cat# 2697, RRID:AB_2079382), IKKα (Cell Signaling Technology Cat# 11930, RRID:AB_2687618), Cleaved-caspase8 (Cell Signaling Technology Cat# 8592, RRID:AB_10891784), Cleaved-caspase3 (Cell Signaling Technology Cat# 9661, RRID:AB_2341188), and GAPDH (Cell Signaling Technology Cat# 5174, RRID:AB_10622025). Ninj1 (Bioss Cat# bs-11105R) was obtained from Bioss, and iNOS (GeneTex Cat# GTX130246, RRID:AB_2886221) antibodies were obtained from GeneTex. Western blotting was performed as previously described [12, 23].

### Reverse transcription-quantitative PCR (RT-qPCR)

Total RNA was extracted from BV2 microglial cells and mouse retinal tissues using the RN002 RNA-Quick Purification Kit (ESscience). cDNA was synthesized using PrimeScript RT Master Mix (TaKaRa). Quantitative amplification of target genes was performed using the Light Cycler 480 Real-Time PCR System with software version LCS480 1.5.1.62. The mRNA levels of the target genes were normalized to the GAPDH level. Primer sequences for target genes are listed in Additional file 6: Table S1.

### Molecular docking

Molecular docking verification of potentially important active components and their key targets was performed as follows: First, the three-dimensional structure file of Ninj1 was quoted from the RCSB protein database (http://www.rcsb.org/), and the crystal structure of NInj1 was synthesized, including removing solvent molecules and ligands, adding polar hydrogen, and energy initialization. The MM2 force field was optimized for the three-dimensional structure of the chemical composition, and the number of rotatable keys was set. The molecular structures of DMHCA were retrieved from PubChem Compound (https://pubchem.ncbi.nlm.nih.gov/). The molecular docking software AutoDock Vina was used for molecular docking scoring, and the scoring threshold was set as − 7 kcal/mol.

### Statistics

We used one-way analysis of variance (ANOVA) followed by Tukey's multiple comparison tests among multiple groups and calculated statistical significance, and unpaired Student's* t*-test between two groups. A *P* value < 0.05 was considered statistically significant. Statistical figures and calculations were performed using GraphPad (https://www.graphpad.com/).

## Results

### Impacts of systemic DMHCA treatment on the general physiological state

**Fig. 1:**
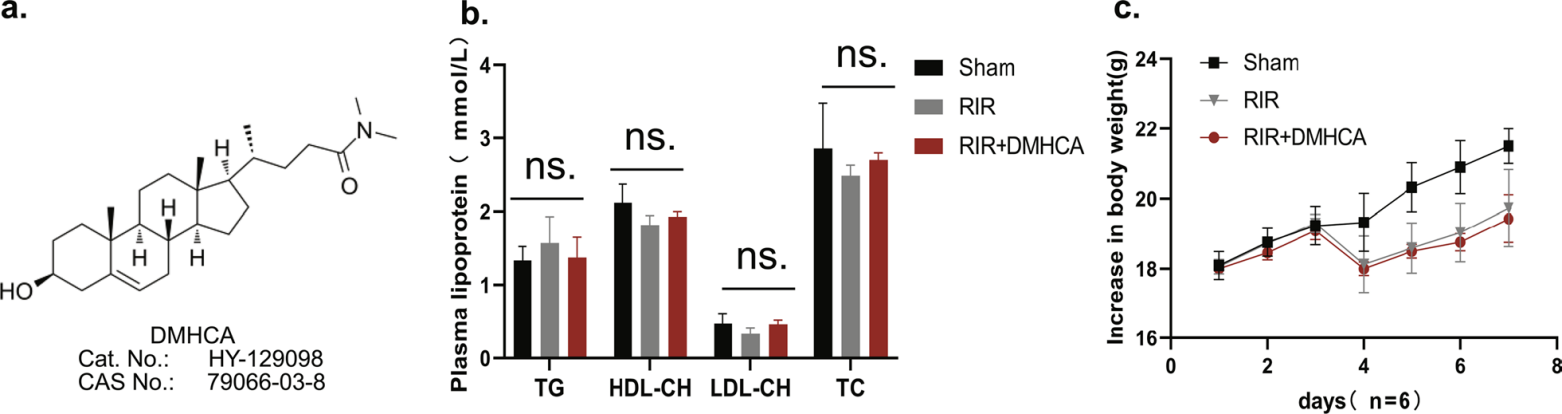
Security evaluation of DMHCA systemic administration on body weight and serum lipid levels in mice. **a** Molecular formula of DMHCA. **b** Serum lipid level in the Sham, RIR, and RIR + DMHCA treatment groups. Detection of triglycerides (TG), total cholesterol (TC), high-density lipoprotein (HDL) cholesterol and low-density lipoprotein (LDL) cholesterol concentrations. Sham, control C57BL/6J with sham operation; RIR, retinal ischemia/reperfusion injury model mice; RIR + DMHCA, DMHCA treatment with RIR mice; *n* = 3–6. ns, *P* > 0.05. **c** Body weight in sham and RIR group: mean C57BL/6 with normal feed and DMHCA group: 80 mg/kg DMHCA of body weight/day daily via oral gavage for 7 days treatment. *n* = 6. Data are expressed as means ± standard deviation

LXR activator plays a key role in cholesterol transport, although is associated with side effects such as hypertriglyceridemia or hepatic steatosis [28]. Therefore, we first assessed the safety of DMHCA (Fig. 1a) on the body weight and serum lipid parameters. Each experimental cycle was seven days and included treatment for the first 3 days; here, we recorded the general physiological data at the end of day 7. In Fig. 1b, no significant differences (*P* > 0.05) were observed in the serum lipid plasma levels, including TC, TG, HDL, and LDL among groups. Additionally, as shown in Fig. 1c, body weight in the sham group continuously and steadily increased under the normal diet. The body weight decreased remarkably after RIR injury, whether in the RIR group or RIR + DMHCA treatment group, and these results were significantly different than those observed in the sham group.

### DMHCA treatment restored IOP-induced RIR injury and RGC death

**Fig. 2:**
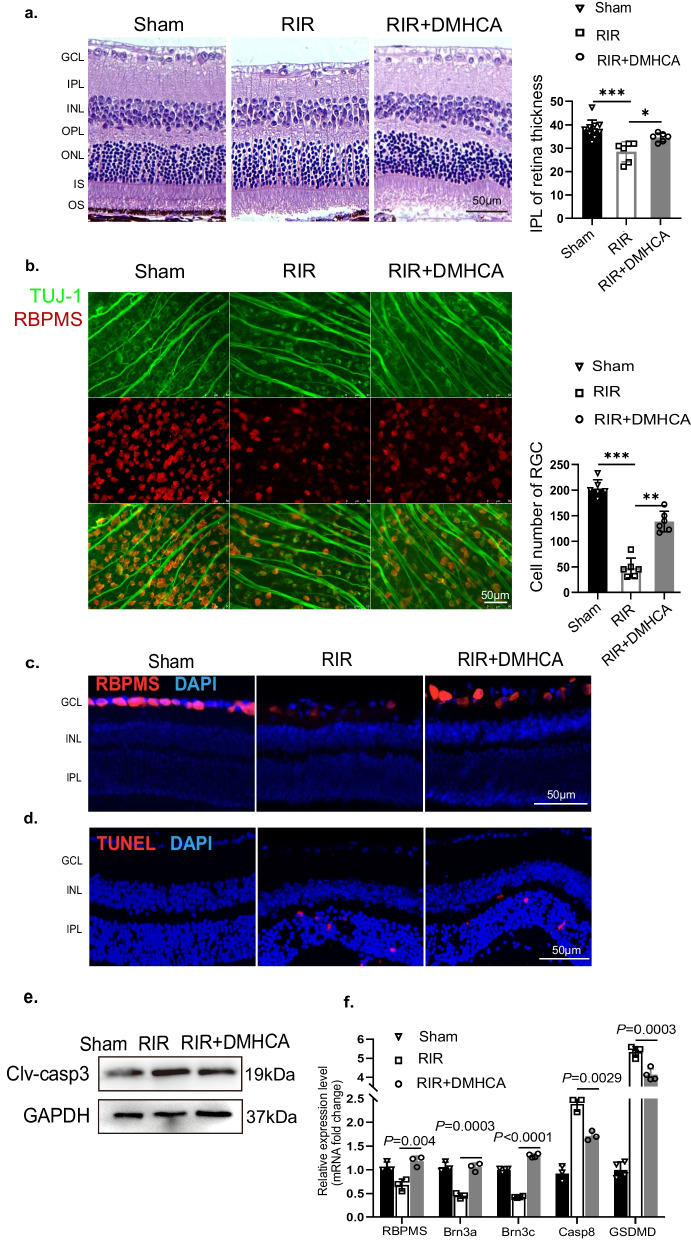
DMHCA treatment significantly attenuate RIR damage. **a** HE staining and quantitative analysis of IPL thickness in retina tissue harvested 3 days post-RIR injury; *n* = 6–10. Scale bar = 50 μm. Sham, control C57BL/6J with sham operation; RIR, retinal ischaemia/reperfusion injury model mice; DMHCA, DMHCA treatment with RIR mice. All in vivo experiments shared this grouping system. **b** Representative immunofluorescence images of anti-RBPMS (red) and anti-TUJ1 (green) labelled RGCs on a flat mount retina. From Sham, RIR, and DMHCA-treated mice at 3 days post-RIR. Quantitative analysis of numbers of RGCs shows a neuroprotection effect of DMHCA; *n* = 6. Scale bar = 50 μm. **c** Representative immunofluorescence images of RGCs in the retinal frozen section. Consistent with images of flat mount retina; *n* > 3. Scale bar = 50 μm. **d** TUNEL staining (red) labelled level of apoptosis in retina, showing an anti-apoptosis effect of DMHCA in vivo; *n* = 3. Scale bar = 50 µm. **e** Western blot analysis of the indicated proteins, Cleaved-caspase3, protein levels were normalized to GAPDH levels. **f** Transcriptional levels of RGC-specific indices, including Brn3a, Brn3c and RBPMS, and programmed cell death-related mRNA, such as caspase 8 and GSDMD, in retinas were detected using qRT-PCR; *n* > 3

We created an IOP-induced RIR injury animal model to mimic the pathogenetic process of acute ischemic retinopathy to evaluate the therapeutic efficacy of DMHCA in vivo. Various cell types are harmed by RIR injury, but RGC loss has been the main focus of models. First, RGCs' dendrites were damaged, displaying a reduction in IPL thickness, and then the soma of the RGCs was found to be damaged, displaying a reduction in the number of ganglion cells (GCL). In order to compare the RIR-induced damage on neurons, these histological parameters (IPL thickness and cell quantity in GCL) can be quantified. Retinas affected by RIR damage showed a substantial reduction in IPL thickness (Fig. 2a). This RIR-induced IPL decrease was improved in the DMHCA-treated retina. RGCs were labelled with RBPMS and β-tubulin-III (Tuj-1) staining in the retina. We quantitatively assessed RGCs in retinal flatmounts (Fig. 2b) and frozen sections (Fig. 2c). Results consistently revealed that the survival rate of RGCs was decreased in response to RIR injury, which was prevented by DMHCA treatment.

In RIR-induced neuroinflammation, apoptosis is essential. The terminal deoxynucleotidyl transferase dUTP nick end labelling (TUNEL)-positive cells and the expression of clv-casp3, an executor protease of apoptosis, were less prevalent in the DMHCA-treated retina than in the RIR counterpart (Fig. 2d) (Fig. 2e). Although we found that DMHCA could reduce RIR-induced RGC loss, the ONL was the primary site where TUNEL positive cells were observed. The TUNEL results were consistent with those of Lam, Chen, and HU [29–31], indicating the induction of apoptosis in RIR retina, the mechanism of which is still unclear.were observed in the GCL.

Additionally, transcriptional levels of RGC-specific indices, including Brn3a, Brn3c, RBPMS, and PCD-related mRNA, such as caspase 8 and GSDMD, were used as indices of nerve injury (Fig. 2f). These results suggest a neuroprotection role of DMHCA.

### Treatment with DMHCA decreased inflammation and inhibited the NF-κB signalling pathway

**Fig. 3:**
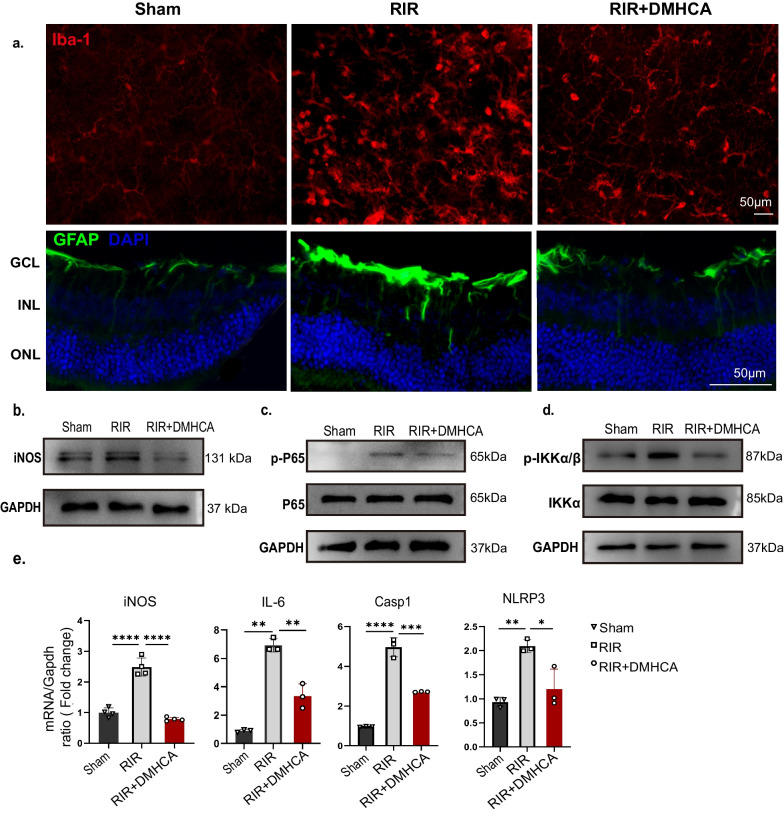
DMHCA decreases inflammation and inhibits NF-κB signalling pathway in acute RIR mice. **a** Representative immunofluorescence images of Iba-1 (red, labelled activation microglia) and GFAP (green, labelled activation macroglia). **b**–**d** Representative western blotting of a pro-inflammatory microglial marker (iNOS) and NF-KB pathway marker (p-P65, P65, p-IKK, and IKK) in retinas. Protein levels were normalized to GAPDH levels. **e** mRNA expression levels of clinical inflammation markers, iNOS, IL-6, caspase 1, and NLRP3 in retinas were measured using qRT-PCR

Resident glial cell activation was observed in RIR injury throughout the process; hence we immunolabelled the glial cells in retina cross-sections with GFAP and Iba-1. GFAP, a Müller cell and astrocyte activation marker, showed higher staining in the RIR group. The fluorescence intensity was reduced in the RIR + DMHCA group (Fig. 3a), indicating that DMHCA alleviated the activation of Müller cells and astrocytes following RIR injury. Microglia play an important role in the surveillance and maintenance of neuronal functionality. When detected via immunostaining, the retinal Iba-1 positive cells in the RIR group were more abundant than equivalent sham retinas, which was one sign of microglia activation.

In contrast, the number of RIR-induced Iba-1^+^ cells was remarkably attenuated by DMHCA (Fig. 3a). Furthermore, in morphology, RIR-induced Iba-1^+^ cells showed a larger soma with shorter and thicker branching processes than their sham counterparts, a phenotype characteristic of the activation stage. When compared with RIR + DMHCA group, Iba-1^+^ cells restore a morphological feature of the resting stage, similar to the sham group. Consistently, DMHCA reduced the protein abundance of iNOS, a marker of the pro-inflammatory microglial phenotype (Fig. 3b).

Since activation of NF-κB signalling pathway has been implicated in promoting neurotoxicity and neuroinflammation in CNS tissue ischemia [32], we explored the potential role of the NF-κB signalling in IOP-induced retinal damage. We examined the activation of an essential protein of the NF-κB pathway and observed that it was significantly inhibited by DMHCA (Fig. 3c, d). To investigate the level of inflammation, we measured the reportedly increased inflammatory mediators in RIR at the retinal levels, such as *iNOS, IL-6, Caspase-1,* and *NLRP3.* In all measures, DMHCA suppressed the level of these cytokines (Fig. 3e).

### *Generation of single-cell transcriptome atlas from sham, RIR, and RIR* + *DMHCA-treated murine retina*

To determine the mechanisms underlying the therapeutic effects of DMHCA, we conducted scRNA-seq of the pooled retinal cells taken from the retinas of sham, RIR, and RIR + DMHCA-treated mice.

**Fig. 4:**
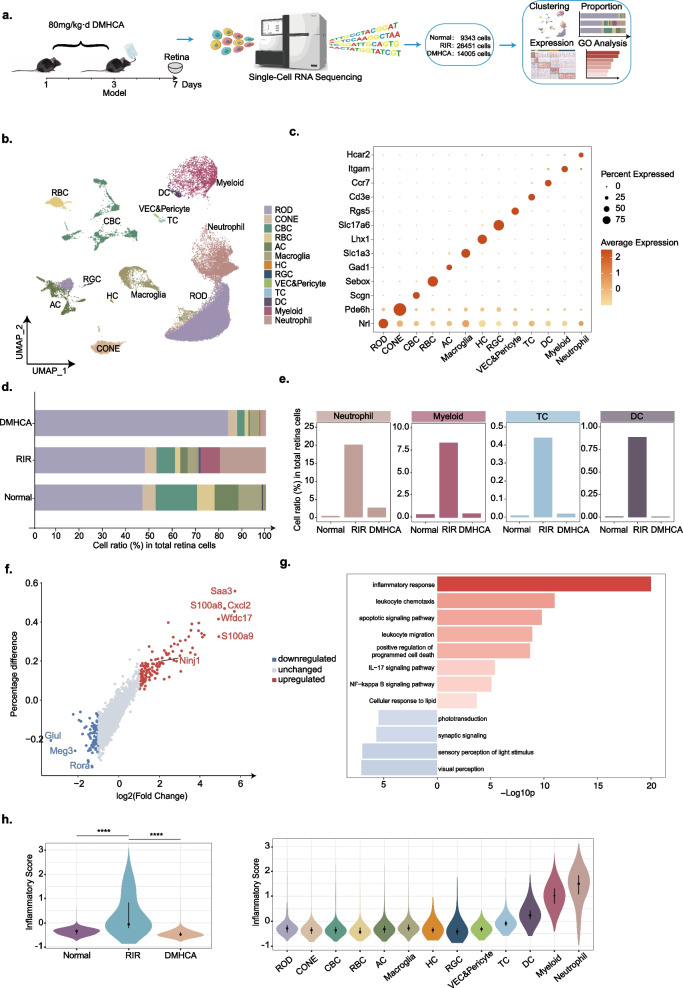
Study design and scRNA-seq analysis of sham, RIR, DMHCA-treated murine retinal cells. **a** Experimental design for scRNA-seq. Retinal samples were collected from sham mice, RIR mice, and DMHCA-treated RIR mice. Each sample was collected from three mice. The gene expression data of the samples were obtained via scRNA sequencing for subsequent single-cell analysis. **b** UMAP clustering of pooled retinal cells coloured according to clusters. **c** Dot plot showing the expression of specific marker genes in each cell type. **d** Bar charts showing proportions of each cell type across the three samples derived from scRNA-seq data. **e** Cell ratio of immune to total retinal cells in the three samples. **f** Dot plot showing the DEGs between the RIR vs. sham comparison group. Red, blue, and grey dots indicate the up-regulated, downregulated, and unchanged DEGs, respectively. **g** Representative GO and KEGG pathway analyses of DEGs of retinal cells in the RIR vs. sham comparison group. Red and blue bars indicate up- and downregulated DEGs, respectively. **h** Violin plot of inflammatory response scores for each sample and cell type. Within each violin plot, middle lines indicate median values, and the line ranges from the 25th to the 75th percentile. Significance was calculated using a two-sided Wilcoxon test as implemented using the function "compare_means" with default parameters; ****P < 0.0001

After quality assurance, we collected high-quality cells totaling 49,799 from the sham sample, 26,451 from the RIR sample, and 14,005 from the sample that had been treated with DMHCA (Fig. 4a). Rods, cones, cone bipolar cells (CBC), rod bipolar cells (RBC), amacrine cells (AC), macroglia, horizontal cells (HC), RGCs, vascular endothelial cells (VEC) and pericytes, T cells (TC), dendritic cells (DC), monocyte macrophage and microglia (Myeloid), and neutrophils were among the cell populations that were distinguished based on the expression of known markers (Fig. 4b, c Additional file 2: a, b). Astrocytes and Müller glia were grouped together in the macroglia population because of the transcriptional overlap. The myeloid group was extracted and further analysed. Although the cluster distributions among the three groups seemed comparable, there were differences in the proportions of single clusters. A considerable number of immune cells appeared after RIR injury and were greatly reduced following DMHCA treatment (Fig. 4d, e; Additional file 2: c).

To explore the retinal cellular response to RIR and DMHCA treatment, we first performed DEG analysis among the three groups (Fig. 4f; Additional file 3: a; Additional file 7: Table S2, Additional file 8: Table S3). Subsequent GO analysis showed that pathways related to immune response, inflammation, and apoptosis were up-regulated during RIR but downregulated by DMHCA treatment, while pathways associated with normal visual functions and maintenance of retinal homeostasis exhibited the opposite trend (Fig. 4g; Additional file 3: b; Additional file 9: Table S4, Additional file 10:  Table S5, Additional file 11: Table S6, Additional file 12: Table S7).

The activity of the inflammatory pathway and overexpression of the specifc genes after RIR showed that RIR induced the inflammatory response. To quantify the severity of these responses enhanced by RIR injury, we next calculated the inflammatory scores in the three samples and found that the retinas of RIR showed an elevation in the inflammatory response score, with neutrophils exhibiting the highest inflammatory response score. Correspondingly, inflammatory responses were suppressed by DMHCA treatment (Fig. 4h). These results suggest that DMHCA suppressed inflammatory responses induced by RIR.

### DMHCA suppressed inflammation in microglia and regulated the expression of Ninj1

Microglia are critical players in glaucoma development because activated microglia exacerbate RGC degeneration through pro-inflammatory and oxidative stress pathways and increase expression of complement molecules [33]. Therefore, we explored the anti-inflammatory effect of DMHCA on microglia [24].

**Fig. 5:**
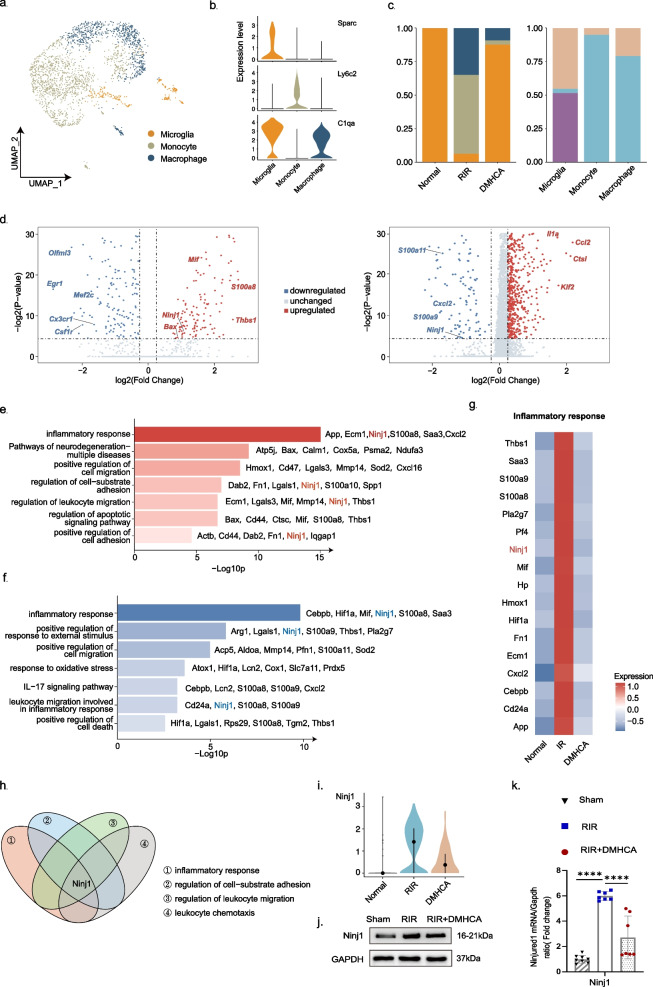
Effects of DMHCA on microglia after RIR and Ninj1 activation. **a** UMAP clustering of myeloid cells. **b** Violin plot showing the expression of the marker genes for each cell type. **c** Bar plots showing cell abundances across myeloid for the sham, RIR, and RIR + DMHCA groups. **d** Dot plot showing the DEGs between the microglia of the RIR vs. sham and RIR + DMHCA vs. RIR comparison groups. Red, blue, and grey dots indicate the up-regulated, downregulated, and unchanged DEGs, respectively. **e**, **f** Representative GO and KEGG pathway analyses of up-regulated DEGs of microglia in the RIR vs. sham comparison group and downregulated DEGs of microglia in the DMHCA vs. RIR comparison group. Red and blue bars indicate up- and downregulated DEGs, respectively. **g** Heatmap showing the expression of enriched genes matched to the GO term inflammatory response. **h** Venn diagram showing the intersection of the genes enriched in GO terms related to inflammation from the previous GO and KEGG pathway analyses of up-regulated DEGs of microglia in the RIR vs. sham comparison group. **i** Violin plot showing the expression of Ninj1 in each group. **j** Western blot analysis of the indicated proteins, Ninj1; protein levels were normalized to GAPDH levels. **k** Transcriptional levels of Ninj1 in retinas were detected using qRT-PCR; *n* = 7

We first extracted the myeloid population and re-clustered the cells into monocytes (Ly6c2 +), macrophages (C1qa +), and microglia (Sparc + C1qa +) (Fig. 5a–c). To identify the underlying mechanism through which DMHCA alleviates RIR-induced stress on the retina, we performed DEG analysis in microglia across the three groups (Fig. 5d; Additional file 13: Table S8, Additional file 14: Table S9) and found that inflammatory gene expression was elevated after RIR injury and reduced in the treatment group. Subsequent GO analysis showed that pathways related to inflammation, immune cell chemotaxis, and apoptosis were up-regulated during RIR injury but downregulated by DMHCA treatment (Fig. 5e, f, g; Additional file 15: Table S10, Additional file 16: Table S11). The pathways associated with the inherent immune function of microglia exhibited the opposite trend (Additional file 3: c, d).

Among the DEGs, we observed that a new gene, *Ninj1*, played a role in several GO pathways associated with inflammatory response, cell migration, and cell adhesion (Fig. 5h). The expression of Ninj1, primarily in myeloid cells and neutrophils, was significantly up-regulated in the RIR group and downregulated after DMHCA treatment (Fig. 5i). As an adhesion molecule, Ninj1 reportedly regulates macrophage function in endotoxin-mediated inflammation and is engaged in the immune responses triggered by cellular infection or stress [34]. Consistent with this notion, our in vivo findings demonstrated that up-regulated Ninj1 was associated with RIR injury and that DMHCA diminished the increase in Ninj1 expression (Fig. 5j, k), suggesting that DMHCA regulates Ninj1 during the development of RIR-induced RGC death.

### Comprehensive cell–cell communication landscape across the three samples

**Fig. 6:**
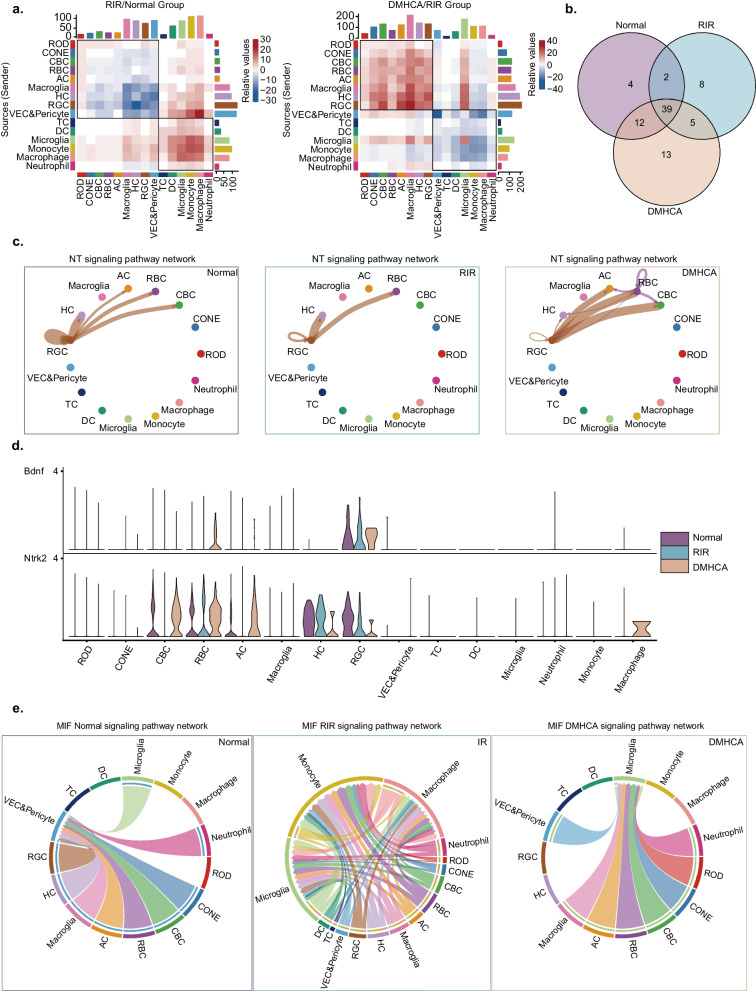
Cell–cell communication landscape in Sham, RIR and DMHCA group. **a** Heatmap showing the number of inferred interactions between the cell types analysed in the RIR vs. sham and RIR + DMHCA vs. RIR comparison groups. **b** Venn diagram showing the number of inferred signalling pathways shared across the three groups. **c** Circle plot showing cell–cell interactions via NT signalling across the three groups. **d** Violin plot showing the expression of the ligand–receptor pairs implicated in NT signalling across the three groups. **e** Chord plot showing cell–cell interactions via the MIF signalling pathway across the three groups

To explore the cell–cell interactions between the three groups, we used CellChat to visualize the communication networks between distinct cell types. Interactions between resident retinal cells were generally downregulated, while immune cell populations exhibited a variable elevation in cell–cell communications with all other cell types in the ischemia–reperfusion retina. RIR + DMHCA-treated group showed an inverse trend, except for microglia among the immune cell subsets (Fig. 6; Additional file 4: a).

Additionally, signalling pathways were identified. The three groups shared several common pathways (Fig. 6b), including the neurotrophin (NT) and migration inhibitory factor (MIF) signalling pathways. The NT signalling pathway can enhance neuronal survival and regeneration and exert a protective effect against RGC apoptosis in the glaucomatous model [35, 36]. As the network indicated, retinal neuronal interactions through the NT signalling pathway were reduced in the RIR group and increased following DMHCA treatment (Fig. 6c, d). Macrophage MIF coding a pro-inflammatory cytokine and the MIF-mediated signalling pathway have been implicated in various inflammatory diseases and disorders [37]. Correspondingly, cell–cell interactions and related ligand–receptor gene expression were markedly up-regulated in the ischemia–reperfusion retina and substantially downregulated when the ischemia–reperfusion retina was exposed to DMHCA treatment (Figs. 6e; Additional file 4: b).

The IGF and CX3C signalling pathways were found in normal and DMHCA-treated retina, while not significant in the RIR group (Additional file 4: c, Additional file 5: d, e, f). In particular, IGF is also a neurotrophic peptide in the CNS, where it reduces neuronal death and augments synaptic plasticity [38]. The CX3CL1–CX3CR1 axis has been implicated in regulating microglial homeostasis, and suppressing microglial activation [39]. Several signalling pathways were exclusive to the RIR + DMHCA group, including EphrinB (EPHB), reelin (RELN), and glial cell line-derived neurotrophic factor (GDNF) signalling pathways. EPHB and RELN-mediated signalling have a major role in RGC axonal targeting [39, 40]. GDNF prevents post-injury RGC death by downregulating extracellular glutamate levels [41].

### Overexpressed *Ninj1* reversed the effects of DMHCA via NF-kB pathway regulation

**Fig. 7:**
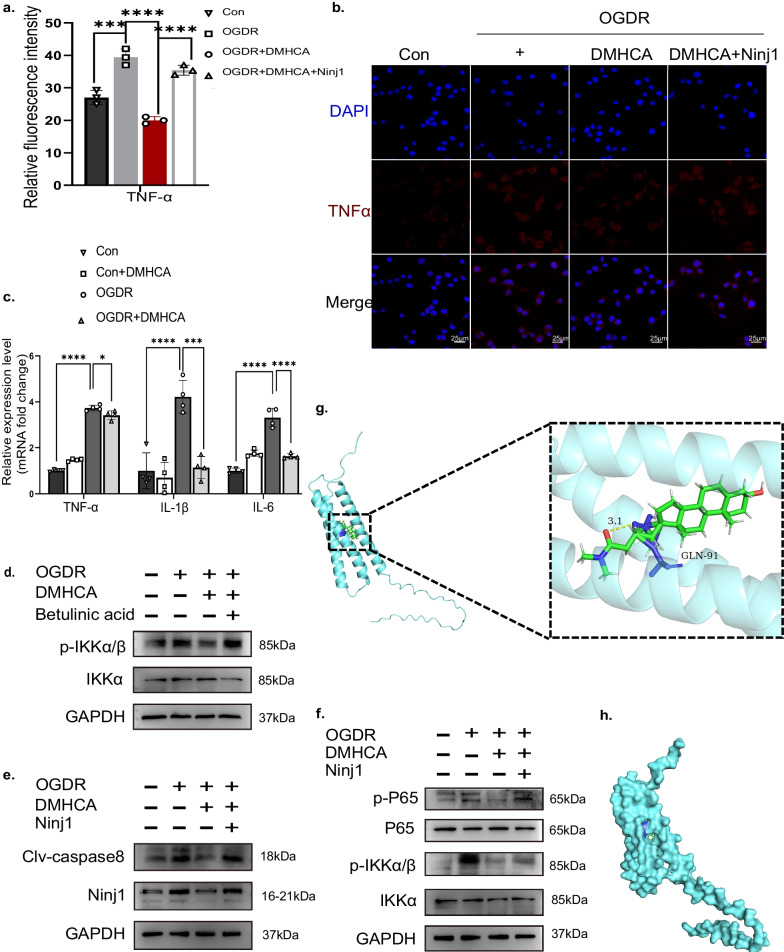
Overexpressed Ninj1 reverted the anti-inflammation effect of DMHCA via regulating NF-κB pathway in vitro. **a, b** Immunofluorescence of TNFα and relative fluorescence intensity among groups (*n* = 3). Scale bar: 25 μm. **c** The mRNA expression levels of TNFα, IL-1β and IL6 in BV2 cells were measured with RT-qPCR (*n* ≥ 3). **d, e, f** Representative Western blots of apoptosis markers (Clv-casp8), Ninj1, NF-kB pathway markers expressions in BV2 cells. **g** Cartoon representation, overlay of the crystal structures of small molecule compound DMHCA and its targets Ninj1 were illustrated. **h** Molecular docking verification of DMHCA and Ninj1. Three-dimensional structures and hydrogen bond of the binding pockets were showed

To explore the underlying mechanism of DMHCA stabilizing microglia during OGD/R-induced inflammation, we detected PCR, western blot, and immunofluorescence (Fig. 7a–c, e). These results showed that TNFα, IL1β, IL6, and cleaved caspase-8 were elevated after OGD/R, whereas DMHCA reversed these changes, suggesting that DMHCA can stabilize the microglia against inflammation.

By reducing the ratio of p-NF-kB p65/NF-kB p65 and p-IKK/IKK in comparison to those in the OGD/R group, DMHCA also drastically decreased the NF-kB signaling pathway and Ninj1, which could be restored by Ninj1 gene overexpression (Fig. 7e, f). We treated microglia with betulinic acid, a transcriptional activator of the NF-kB pathway (S3603; Selleck Chemicals, USA), to confirm the anti-inflammatory effects of DMHCA via the NF-kB pathway, and discovered that it counteracted the inhibition of RIR + DMHCA group on the key molecule of the NF-kB signalling pathway (Fig. 7d). These results suggest that the NF-kB pathway was controlled by over-expressed Ninj1, which negated the anti-inflammatory effects of DMHCA.

To evaluate the affinity of DMHCA for Ninj1, we did the molecular docking experiment. The binding poses and interactions of DMHCA and the Ninj1 protein were gained using Autodock Vina v.1.2.2, and binding energy for each interaction was calculated (Fig. 7g, h). Results showed that DMHCA bound to its target Ninj1 through a visible hydrogen bond and strong electrostatic interactions. For Ninj1, DMHCA had a low binding energy of − 6.6 kcal/mol, indicating a highly stable binding ability.

## Discussion

We explored the intraocular therapeutic efficacy, mechanism, and systemic safety of short-term DMHCA treatment in developing IOP-induced retinal damage. Our main findings are as follows: (1) Prophylactic treatment with DMHCA prior to injury effectively reduced neuroinflammation and RGC apoptosis, thus restoring the retinal structure in retinal ischemia–reperfusion; (2) DMHCA attenuated microglia-mediated inflammation and polarization by inhibiting activation of the NF-kB signalling pathway; (3) DMHCA-mediated anti-inflammation and anti-apoptosis effects were, at least partly, due to alleviation of Ninj1, and further support came from the overexpression of Ninj1 largely abrogated the protection effects of DMHCA by up-regulating the expression of TNFα and clv-caspase8; (4) Short-term (3 days) administration and a high dose (80 mg/kg of body weight/day) of systemic took DMHCA did not increase serum TG or cholesterol levels; furthermore, DMHCA is a safe and effective therapeutic option against RIR injury.

Substantial evidence suggests that retinal ischemia–reperfusion is the main mechanism of glaucoma and the cause of irreversible vision loss globally [42]. Therefore, exploring innovative drugs for RIR treatment is urgent. Many inducible rodent models of RIR injury include clipping the retinal vessels and cautery of extraocular veins [43]. The most frequently used model is the "pressure-induced RIR model" [12], adopted in the present study. Research has revealed that the secretion of TNFα increases independently following RIR injury, which leads to RGC damage and optic nerve degeneration, primarily through originating its downriver apoptotic signal cascade [44, 45]. In the retina, TNFα is primarily secreted by microglia [6]. Overmuch release of cytokines, chemokines, and other inflammatory mediators by necrotic cells in the later stages further intensifies the inflammatory level. Microglia are intrinsic immunological cells present in the retina; owing to their toxic or protective effects in different contexts, we used the anti-inflammatory effect of microglia as an important evaluation index. We confirmed that DMHCA ameliorated the microenvironment of inflammation in microglia by downregulating the levels of pro-inflammatory cytokines, TNFα, IL1β, and IL6, and inhibiting the expression of apoptosis markers such as cleaved caspase 8, under OGD/R-induced inflammation. Similarly, several studies have shown that LXR activation decreases the expression of a few genes that mediate inflammation, such as *iNOS*, *COX2*, *MMP9*, *IL-1β,* and *IL-6* [46]. In addition, microglia can rapidly change states in response to various stimuli in the microenvironment. Accompanying the inflammatory activity, we also found that the expression of microglial canonical markers, such as Serpine2, Olfml3, Gpr34, and Sparc, was remarkably reduced per the scRNA-seq results of our RIR group (Additional file 3: e), a phenotype reported in some CNS damages or diseases related to neuroinflammation [47, 48]. The expression of these genes was partially up-regulated in the RIR + DMHCA group, indicating that microglia had switched to a more homeostatic "resting" state. These results imply that DMHCA was instrumental in ameliorating microglia-mediated inflammation, which provides evidence of the beneficial neuroprotective effects of DMHCA in the RIR model.

Next, we explored the specific pathomechanism underlying the anti-inflammatory effects of DMHCA. Previous investigations have indicated that DMHCA might inhibit inflammation and cholesterol accumulation in glomeruli through LXR [49]. Additionally, in immune cells, particularly macrophages, LXRs inhibit pro-inflammatory gene expression [50]. These findings prompted us to investigate the transcription factor activity of LXR. We used PYSCENIC to compare the transcription factor activity between the RIR and DMHCA-treated groups and found that LXRα activity was not detected, while LXRβ surprisingly showed lower transcription factor activity in the DMHCA-treated retina (Additional file 17: Table S12). We then assessed the expression of the LXR-controlled genes using the scRNA-seq data and showed that both Abca1 and ApoE expression were downregulated following DMHCA treatment (Additional file 18: Table S13). Silico's analysis confirmed that the LXR pathway was not activated as a hinge in the retina through DMHCA treatment, which is consistent with previous findings [51].

After RIR injury and therapy, we evaluated the global retinal landscape under homeostatic settings using our previously published single-cell transcriptome datasets [13]. We determined the proportionate changes in 13 cell populations. Ninj1 was enriched in several inflammatory, leukocyte migration, and chemotaxis-related pathways in the scRNA-seq data used in our studies, suggesting that Ninj1 plays a role in immune cell recruitment and the subsequent inflammatory damage in injury pathogenesis brought on by ocular hypertension. In vivo and in vitro, we noticed a noticeably higher expression of Ninj1 after inflammation. In line with this, Xiao [52] found that the levels of Ninj1 mRNA in circulating leukocytes from injured persons were significantly increased (1.87-fold). In endotoxin-mediated inflammation, diabetes, and atherosclerosis, Ninj1 controls macrophage activity [34].

As no specific Ninj1 activator exists, we utilized genetic manipulations to overexpress Ninj1 and explore its distinct role in OGD/R injury. Our result implied that compared with OGD/R, treatment with DMHCA decreased Ninj1 levels. Additionally, microglia-specific overexpression of Ninj1 increased inflammatory gene expression and apoptosis in the OGD/R model in vitro (Fig. 7c). Similarly, Ninj1-overexpressed macrophages in Hwang's study [34] showed notably higher levels of inflammatory-related proteins and proangiogenic factors. Moreover, the authors found that inhibiting Ninj1 reduced these inflammatory mediators. To analyse the binding affinities and the possibility of interaction between DMHCA and Ninj1, AutodockVina 1.2.2, a silico protein–ligand docking software, was utilized. The data support a strong ability of DMHCA to incorporate into and directly bind with the membrane protein, Ninj1, which may contribute to some of the observed pleiotropic effects. DMHCA possesses chemical properties of amphipathicity owing to the synthetic oxysterol with a shortened sidechain and amide moiety. Similar to other oxysterols, DMHCA can be incorporated into the exofacial leaflet of the membrane, thereby directly modifying the functions of integral and membrane-associated proteins [53]. This supports our hypothesis that DMHCA can directly interact with Ninj1, indicated by preliminary data. The underlying mechanisms remain to be elucidated. Despite our efforts to determine the role of Ninj1 in hypoxia and glucose-free induced microglial activation in vitro, other cells besides microglia might have contributed to the observed. NF-κB (p65) is an important transcriptional factor that regulates IL-1β and several inflammatory cytokines, while NF-κB activation plays a pivotal role in RIR-induced neuroinflammatory processes secondary to RGC damage [29, 54]. Our scRNA-seq results indicate that the NF-κB signalling pathway was enriched in global RIR retinal cells and the immune cell subset, including the myeloid and neutrophil populations. NF-κB pathway regulation was further confirmed by the decrease in the NF-κB pathway protein levels observed following DMHCA treatment compared with those of the RIR model group both in vivo and in vitro. Previous preclinical and clinical studies have suggested that the NF-κB pathway has an anti-inflammatory effect on inflammatory diseases, including dry eye syndrome and glaucoma [55, 56], which further affirms the potential clinical value of DMHCA for the treatment of glaucoma.

In summary, this study showed that DMHCA significantly attenuated RIR-induced neuroinflammation, such as alleviating the activation of microglia and decreasing the levels of inflammatory mediators, thereby hindering apoptosis in RIR-injured mice. Furthermore, DMHCA ameliorated neuroinflammation by suppressing Ninj1 and NF-κB pathway activity. However, genetic overexpression of Ninj1 reversed the anti-inflammatory and anti-apoptotic effects of DMHCA in the microglia cell line. These findings suggest that Ninj1 plays an important role in the mechanisms of RIR injury and that DMHCA is a promising potential treatment against RIR through Ninj1 inhibition.

## Supplementary Information

**Additional file 1.** Expression vector cloning and transfection effect.

**Additional file 2.** UMAP clustering, feature plot and cell ratio of whole retinal cells. **a** The UMAP clustering of pooled retinal cells, colored by groups. **b** UMAP plot showing the canonical markers for retinal cells and immune cells. **c** The fold change (FC) of cell ratios in different cell types between the RIR/Sham, DMHCA/RIR comparison group. The numbers on the right indicate the Log2FC value of the cell ratios.

**Additional file 3.** DEG plot and GO analysis of whole retinal cells and microglia subsets. **a** Dot plot showing the DEGs between the DMHCA/RIR comparison group. Red, blue and grey dots indicate the up-, down-regulated and unchanged DEGs, respectively. **b** Representative GO and KEGG pathway analysis of DEGs of retinal cells in the DMHCA/RIR comparison group. Red and blue bars indicate up- and down-regulated DEGs, respectively. **c** Representative GO and KEGG pathway analysis of downregulated DEGs of microglia in the RIR/Sham comparison group. **d** Representative GO and KEGG pathway analysis of upregulated DEGs of microglia in the DMHCA/IR comparison group. **e** Dotplot showing the expression of marker genes between the Sham, RIR and DMHCA groups.

**Additional file 4.** Signaling pathways of cell-cell interactions. **a** Heatmap showing the strength of inferred interactions between the cell types analyzed in the RIR/Sham and DMHCA/RIR comparison group. **b** Violin plot showing the expression of ligand-receptor pairs implicated in the MIF signaling across the three groups. **c** Circle plot showing cell-cell interactions via IGF signaling in the Sham and DMHCA group.

**Additional file 5.** Signaling pathways and expression of ligand-receptors of cell-cell interactions. **a** Violin plot showing the expression of ligand-receptor pairs implicated in the IGF signaling across the three groups. **b** Circle plot showing cell-cell interactions via CX3C signaling in the Sham and DMHCA group. **c** Violin plot showing the expression of ligand-receptor pair implicated in the CX3C signaling across the three groups.

**Additional file 6. Table S1.** The primers used for the amplification of the target genes.

**Additional file 7. Table S2.** Table of DEGs between sham and RIR groups.

**Additional file 8. Table S3.** Table of DEGs between RIR and DMHCA groups.

**Additional file 9. Table S4.** Table of GO analysis of upregulated DEGs in RIR group compared to sham group.

**Additional file 10. Table S5.** Table of GO analysis of downregulated DEGs in RIR group compared to sham group.

**Additional file 11. Table S6.** Table of GO analysis of downregulated DEGs in DMHCA group compared to RIR group.

**Additional file 12. Table S7.** Table of GO analysis of upregulated DEGs in DMHCA group compared to RIR group.

**Additional file 13. Table S8.** Table of DEGs of microglia between sham and RIR groups.

**Additional file 14. Table S9.** Table of DEGs of microgliabetween RIR and DMHCA groups.

**Additional file 15. Table S10.** Table of GO analysis of DEGs of microglia in RIR group compared to sham group.

**Additional file 16. Table S11.** Table of GO analysis of DEGs of microglia in DMHCA group compared to RIR group.

**Additional file 17. Table S12.** Table of regulon activity of inferred transcription factors between DMHCA and RIR groups.

**Additional file 18. Table S13.** Table of average gene expression between RIR and DMHCA groups.

## Data Availability

The data that support the findings of this study are available from the corresponding author upon request. The scRNA-seq data are deposited in the Genome Sequence Archive in BIG Data Center, Beijing Institute of Genomics (BIG, https://ngdc.cncb.ac.cn/gsa/), Chinese Academy of Sciences, under the Project Accession No. PRJCA013579. The data are available from the corresponding author on reasonable request.

## Abbreviations

RIR: Retinal ischemia–reperfusion
IPL: Inner plexiform layer
NS: Normal saline
IOP: Intraocular pressure
CNS: Central nervous system
RGC: Retinal ganglion cell
NF-κB: Nuclear factor kappa B
scRNA-seq: Single-cell RNA sequencing
OGD/R: Oxygen and glucose deprivation/reoxygenation
*Ninj 1*: Nerve injury-induced protein 1
TNFα: Tumour necrosis factor-α
IL: Interleukin
UMAP: Manifold Approximation and Projection
KEGG: Kyoto Encyclopaedia of Genes and Genomes
TF: Transcription factor
ANOVA: Analysis of variance
GCL: Ganglion cell layer
TC: T cells
DC: Dendritic cells
CBC: Cone bipolar cells
RBC: Rod bipolar cells
AC: Amacrine cells
HC: Horizontal cells
VEC: Vascular endothelial cells
NT: Neurotrophin
EPHB: EphrinB
RELN: Reelin
GDNF: Glial cell line-derived neurotrophic factor
