# Supplementary material for: N,N-Dimethyl-3β-hydroxycholenamide attenuates neuronal death and retinal inflammation in retinal ischemia/reperfusion injury by inhibiting Ninjurin 1
Source: J Neuroinflammation. 2023 Apr 7;20:91. doi: 10.1186/s12974-023-02754-5 (PMC10082498; doi:10.1186/s12974-023-02754-5)
Supplement: Supplementary file 6 — Additional file 6. Table S1. The primers used for the amplification of the target genes. [file 12974_2023_2754_MOESM6_ESM.doc]

**Table S1:** The primers used for the amplification of the target genes

|  |  |  |
| --- | --- | --- |
| **Gene** | **Forward primer (5'-3')** | **Reverse primer (5'-3')** |
| *RBPMS* | GTACCCAGCGGAGTTAGCG | AAGACAGGTGTGTTGGGCTTT |
| *Brn3a* | TGAGCACAAGTACCCGTCG | GCACGCTATTCATCGTGTGGT |
| *Brn3c* | CGACGCCACCTACCATACC | CCCTGATGTACCGCGTGAT |
| *Caspase8* | TGCTTGGACTACATCCCACAC | GTTGCAGTCTAGGAAGTTGACC |
| *GSDMD* | CGATGGAACGTAGTGCTGTG | TCCTTCCCAACCTGCTGTTG |
| *Ninj1* | ACTGAGGAGTATGAGCTCA | TCCATTACAGGCTTCTGGA |
| *IL-6* | AGTTGCCTTCTTGGGACTGA | TCCACGATTTCCCAGAGAAC |
| *Caspase1* | ACAAGGCACGGGACCTATG | TCCCAGTCAGTCCTGGAAATG |
| *NLRP3* | ATTACCCGCCCGAGAAAGG | TCGCAGCAAAGATCCACACAG |
| *iNOS* | CCCTTCAATGGTTGGTACATGG | ACATTGATCTCCGTGACAGCC |
| *IL-1β* | CTCCATGAGCTTTGTACAAGG | TGCTGATGTACCAGTTGGGG |
| *TNFα* | CAGGCGGTGCCTATGTCTC | CGATCACCCCGAAGTTCAGTAG |
| *GAPDH* | TGACCTCAACTACATGGTCTACA | CTTCCCATTCTCGGCCTTG |
